# Perceived social support and coping style as mediators between resilience and health-related quality of life in women newly diagnosed with breast cancer: a cross-sectional study

**DOI:** 10.1186/s12905-022-01783-1

**Published:** 2022-05-28

**Authors:** Kaina Zhou, Fan Ning, Xiao Wang, Wen Wang, Dongfang Han, Xiaomei Li

**Affiliations:** 1 School of Nursing, Xi’an Jiaotong University Health Science Centre, No. 76 Yanta West Road, Xi’an, 710061 Shaanxi China; 2 Surgical Breast Cancer Department, The Tumor Hospital of Shaanxi Province, No. 309 Yanta West Road, Xi’an, 710061 Shaanxi China; 3 Gynecology Department, The First Affiliated Hospital of Xi’an Jiaotong University, No. 277 Yanta West Road, Xi’an, 710061 Shaanxi China

**Keywords:** Breast cancer, Resilience, Perceived social support, Coping style, Health-related quality of life

## Abstract

**Background:**

Breast cancer may impair health-related quality of life (HRQoL). We examined the mediating roles of perceived social support (PSS) and coping style (CS) in the relationship between resilience and HRQoL in newly diagnosed breast cancer patients.

**Methods:**

Following a cross-sectional design, 431 patients completed a survey at two hospitals in Shaanxi Province, China. Four validated self-report measures assessed HRQoL, psychological resilience, PSS, and CS. A one-sample *t*-test analyzed differences between resilience, PSS, and CS in breast cancer patients and the corresponding norm. Multivariate linear regression analyzed the independent predictors of HRQoL. The mediating roles of PSS and CS between resilience and HRQoL were investigated using structural equation modeling (SEM).

**Results:**

Participants had significantly lower scores for resilience and PSS, and higher scores for the avoidance and resignation CSs than their corresponding norm. SEM analysis showed resilience had significant direct effects on PSS (*B*s: 0.59, 95% CI 0.49, 0.68, *P* = 0.003), CS (confrontation: 0.53 (0.44, 0.62), *P* = 0.001; resignation: − 0.66 (− 0.74, − 0.57), *P* = 0.002), and HRQoL (*B*s range from 0.44 to 0.63, *P* < 0.05). Resilience had significant indirect effects (*B*s range from 0.09 to 0.27), and PSS and CS had significant direct effects on HRQoL (*P* < 0.05).

**Conclusions:**

Newly diagnosed breast cancer patients had lower resilience and PSS, and higher negative CSs, suggesting that PSS and CS mediated the influence of resilience on HRQoL. A multimodal intervention program focusing on PSS and CS might improve the positive influences of resilience on HRQoL in breast cancer patients.

## Background

As a major stressful life event, breast cancer diagnosis and treatment-related adverse effects could cause severe physical and psychological trauma to the patient, and eventually, result in damage to their health-related quality of life (HRQoL) [1–4]. HRQoL refers to an individual’s self-perception of their physical, psychological, and social functioning, and has been regarded as an essential indicator for evaluating the overall therapeutic effect of cancer treatment and the overall functional rehabilitation of patients during their lifetime [5]. However, diseases and various negative stressors affect the HRQoL of breast cancer patients with varying degrees, especially in the field of psychosocial functioning [5]. Thus, the improvement of breast cancer treatment strategies and an increased survival rate has made the psychosocial rehabilitation of patients increasingly important [6].

From the view point of coping with a stressful life event, three personal-related psychosocial factors should be mainly considered. The first factor is resilience, which shows personal ability in adapting and successfully coping with adversity, and changes continuously with the interactions between the individual and the environment [7–9]. The second factor is coping style, which includes confrontation, avoidance, and resignation. It refers to the methods and strategies with personal characteristics adopted by individuals in order to reduce or avoid stress and adapt to the environment [10]. The third factor is perceived social support, which indicates personal understanding and feeling of social support from family members and significant others [11].

During the long-term rehabilitation process of breast cancer, the above three factors are important in predicting HRQoL, respectively [12–15]. However, given the interactions among resilience, coping style and perceived social support, it is not realistic and feasible for health care providers to develop an intervention program with considering only one personal-related psychosocial factor in real clinical context, especially for those patients with newly breast cancer diagnosis. After all, such patients lack the adaptation and coping experiences, and may have more demands of social support from their family members or significant others while confronting with breast cancer. Following further literature reviews, we found that resilience and active coping style have positive effects on the self-reported quality of life in cancer patients [16], and social support and resilience are critical to HRQoL, with social support as a major mediator [17, 18]. Regarding the comprehensive rehabilitation, it is important of higher resilience, satisfied perception of social support from family members or significant others, and positive confrontation with the adverse event to the whole recovery of physical, psychological, and social function, especially in newly diagnosed patients. Unfortunately, the existed studies have not answered the question of whether perceived social support and coping style play mediator roles between resilience and HRQoL in women newly diagnosed with breast cancer.

This study is based on the Neuman Systems Model (NSM), a theoretical framework for nursing that emphasizes the interaction between people and the environment, and the individual’s response to stressors in the environment. The main components of this model include the stressor, the body’s defense, and nursing interventions. When a stressor acts on the body, the body reacts defensively, and the purpose of a nursing intervention is to maintain and restore the balance of the body’s system [19].

**Fig. 1:**
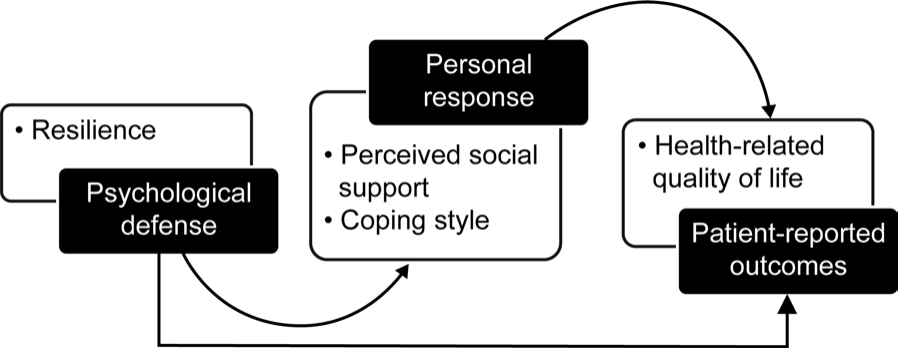
Theoretical framework of the mediating factor model regarding health-related quality of life based on the Neuman Systems Model

The components of the NSM were used as follows. Each woman with breast cancer is an independent system; the disease and its treatment-related factors (stressors) will have various negative effects on the patient (system), and then cause changes in their psychological toughness (body defense). Through comprehensive consideration of the positive role of the patient’s self-defense capabilities and the positive guiding effects of a patient’s personal responses (i.e., CS and PSS), resilience was regarded as the body’s defense, PSS and CS as the personal responses, and HRQoL as the patient-reported outcome (Fig. 1). This model was applied to identify the mediator roles of PSS and CS (i.e., mediator variables) in the relationship between resilience (i.e., independent variable) and HRQoL (i.e., outcome variable).

The purpose of the study is to examine the mediator roles of PSS and CS in the relationship between resilience and HRQoL in women newly diagnosed with breast cancer. There are two hypotheses: (1) resilience, PSS, and CS are independent predictors of HRQoL; and (2) PSS and CS mediate the relationship between resilience and HRQoL. The study’s findings will provide new evidence for identifying the mediator roles of psychosocial factors in the relationship between resilience and HRQoL, and for developing multimodal intervention programs to improve HRQoL in women newly diagnosed with breast cancer.

## Methods

### Design

We used a cross-sectional study design.

### Participants and sampling

Convenience sampling was used to recruit women with breast cancer admitted to the Tumor Hospital of Shaanxi Province or the First Affiliated Hospital of Xi’an Jiaotong University. Inclusion criteria were women newly diagnosed with breast cancer, aged 18 years or older, and Chinese speakers. The exclusion criteria were patients with other comorbid non-breast tumors, breast diseases, or cognitive disorders (screened by a blinded psychiatrist according to the criteria of the Diagnostic and Statistical Manual of Mental Disorders, 5th ed.). The required sample size was estimated based on the metric of 5–10 participants per item in a validated instrument [20] to ensure sufficient power. Considering that the largest instrument used has 36 items, the appropriate sample size should be 180–360 patients.

### Measurements

#### Functional assessment of cancer therapy-breast version 4.0

Patients’ HRQoL was measured by the 36-item Chinese Functional Assessment of Cancer Therapy–Breast version 4.0 (FACT-Bv4.0). Each item is rated on a 5-point Likert scale (0 = *not at all*, 4 = *very much*). The items are divided into five subscales (i.e., social/family well-being [SWB, 7 items], physical well-being [PWB, 7 items], functional well-being [FWB, 7 items], emotional well-being [EWB, 6 items], and breast cancer-specific [BCS] concerns, 9 items) [21]. The FACT-Bv4.0 total score (ranging from 0 to 144) is the sum of the five subscales scores, with a higher score indicating better HRQoL [22]. A validation study of the Chinese FACT-Bv4.0 resulted in higher reliability (Cronbach’s α of the five subscales ranges from 0.59 to 0.85) and satisfactory validity [22]. In this study, the Cronbach’s α for the overall scale was 0.93 and, for each of the five subscales, it was 0.85 (PWB), 0.91 (SWB), 0.86 (EWB), 0.89 (FWB), and 0.70 (BCS).

#### Connor–Davidson resilience scale

The 25-item Chinese Connor–Davidson Resilience Scale (CD-RISC) was used to measure psychological resilience. Responses are indicated on a 5-point Likert-type scale, ranging from 0 (*not true at all*) to 4 (*true nearly all the time*). Items are divided into three subscales—tenacity (15 items), self-reliance (7 items), and optimism (3 items). The total score ranges from 0 to 100, with a higher score reflecting higher resilience [23]. The original English version has good reliability (Cronbach’s α = 0.89) and validity [24]. The Chinese CD-RISC also has high reliability (Cronbach’s α = 0.91) and satisfactory validity [23]. In this study, the Cronbach’s α values were 0.95 (CD-RISC), 0.92 (tenacity), 0.77 (self-reliance), and 0.88 (optimism).

#### Multidimensional scale of perceived social support

The 12-item Chinese Multidimensional Scale of Perceived Social Support (MSPSS) measures perceived support from family, friends, and significant others. Items are rated on a 7-point Likert scale (1 = *very strongly disagree*, 7 = *very strongly agree*). The total score is the sum of all items, ranging from 12 to 84, with a higher score indicating higher PSS [25]. The original English version has good internal reliability and strong factorial validity [26]. The Chinese MSPSS has been well validated in university students (Cronbach’s α = 0.92) [27] and in patients with methadone maintenance treatment (Cronbach’s α = 0.92) [28]. In this study, the Cronbach’s α values were 0.93 (MSPSS), 0.91 (family support, 4 items), 0.90 (friends support, 4 items), and 0.93 (significant others support, 4 items).

#### Medical coping mode questionnaire

The 20-item Chinese Medical Coping Mode Questionnaire (MCMQ) was used to assess the CS of patients with breast cancer. It has three subscales, namely confrontation, avoidance, and resignation, with a higher subscale score indicating more confrontation, avoidance, or resignation, respectively [29]. The original and Chinese MCMQs have satisfactory psychometric properties (Cronbach’s α ranged from 0.69 to 0.76) [29, 30]. In this study, the Cronbach’s α for the three subscales were 0.70 (confrontation, 8 items), 0.72 (avoidance, 7 items), and 0.88 (resignation, 5 items).

### Data collection

Data were collected from September 2020 to July 2021. Patients were instructed to complete the questionnaires by themselves. If the patient had reading or writing difficulties, a trained data collector read the items to the patient and recorded their responses.

### Data analyses

Categorical variables were summarized using frequencies and percentages, while continuous variables were summarized using mean and standard deviation (SD). A one-sample *t*-test was utilized to compare the total score of the CD-RISC and MSPSS, and the three subscales scores of the MCMQ with the corresponding normative data. A multivariate linear regression analysis was performed to identify the influence of resilience, PSS, and CS on HRQoL when controlling for socio-demographics and clinical characteristics.

Structural equation modeling (SEM) was employed using the maximum likelihood bootstrapping method [29] to examine the mediating role of PSS and CS on the relationship between resilience and HRQoL. Standardized direct, indirect, and total effects, and R^2^ with a corresponding 95% bias-corrected confidence interval were estimated based on 1 000 random samples (bootstrapping method subsample) generated by computer [31]. The mediating roles were examined via three stages [32]: (1) significant direct effects of resilience (independent variable), PSS, and CS (mediators); (2) significant direct effects of resilience on HRQoL (outcome variable); and (3) significant indirect effects of resilience and direct effects of PSS and CS on HRQoL. The model fit was examined with χ^2^ value (desired significance *P* > 0.05), adjusted goodness-of-fit index (AGFI; desired value ≥ 0.90), and root mean square error of approximation (RMSEA; desired value < 0.08) [33]. The data were analyzed using SPSS 25.0 and AMOS 21.0 (IBM Corp., Armonk, NY). A value of *P* < 0.05 (two-tailed) was considered statistically significant.

### Ethical statement

The Human Research Ethics Committee of Xi’an Jiaotong University reviewed and approved the study protocol (2019-9800). Written informed consent was obtained from each patient before administering the questionnaire survey. Furthermore, the study conforms to the standards contained in the Declaration of Helsinki, as amended.

## Results

A total of 440 patients met the eligibility criteria and 431 (98.0%) completed the questionnaires; 127 (29.5%) were from the Tumor Hospital of Shaanxi Province, and 304 (70.5%) were from the First Affiliated Hospital of Xi’an Jiaotong University. Nine patients were excluded due to comorbid non-breast tumors (*n* = 3), other breast disease (*n* = 4), and refusal to provide written informed consent (*n* = 2). Table 1 summarizes the socio-demographic and clinical characteristics of the sample, and Table 2 presents the summary results for the four questionnaires used in the data analysis.

**Table 1:** Participants’ socio-demographics, clinical characteristics, and summary results for the four questionnaires (*N* = 431)

	*n* (%)
*Socio-demographics*	
Age (years; mean ± SD^a^; range: 27–77)	48.26 ± 10.40
Education Level	
Primary and below	90 (20.9)
Secondary	278 (64.5)
Tertiary	63 (14.6)
Marital status	
Married	425 (98.6)
Other marital status	6 (1.4)
Has children	
Yes	416 (96.5)
No	15 (3.5)
Employment status	
Unemployed	170 (39.4)
Retired	54 (12.5)
Employed	207 (48.1)
Average monthly income over the past 12 months (Chinese Yuan)	
< 3000	170 (39.4)
3000–6000	194 (45.0)
6001–9000	52 (12.1)
> 9000	15 (3.5)
Place of residence	
Urban	207 (48.0)
Rural	224 (52.0)
Chronic disease (disease course > 6 months)	
Yes	28 (6.5)
No	403 (93.5)
*Clinical characteristics*	
Illness stage	
0–I	134 (31.1)
II	203 (47.1)
III	66 (15.3)
IV	28 (6.5)
Metastasis	
No metastasis	285 (66.1)
Axillary	119 (27.6)
Other metastasis	27 (6.3)
Surgical style	
No surgery	146 (33.9)
Modified radical mastectomy	203 (47.1)
Simple mastectomy	60 (13.9)
Breast conservation	23 (5.3)
Adjuvant therapy	
Conventional chemotherapy	381 (88.4)
Neoadjuvant chemotherapy	26 (6.0)
Radio therapy	151 (35.0)
Endocrine therapy	231 (53.6)
Targeted therapy	47 (10.9)
*HRQoL*^*b*^* (mean* ± *SD)*	
Physical well-being	17.75 ± 4.91
Social/family well-being	17.66 ± 5.95
Emotional well-being	14.29 ± 4.48
Functional well-being	13.24 ± 5.04
Breast-cancer-specific	
Subscales for additional concerns	24.81 ± 3.97
FACT-Bv4.0^c^ Total Score	87.74 ± 18.69
*Resilience (mean* ± *SD)*	
Tenacity	27.51 ± 8.19
Self-reliance	16.84 ± 4.42
Optimism	10.37 ± 3.06
CD-RISC^d^ total score	54.72 ± 14.71
*Perceived social support (mean* ± *SD)*	
Family support	24.16 ± 3.24
Friends’ support	20.76 ± 4.00
Significant others’ support	20.52 ± 4.17
MSPSS^e^ total score	65.45 ± 9.40
*Coping style (mean* ± *SD)*	
Confrontation	19.87 ± 3.73
Avoidance	16.70 ± 1.90
Resignation	9.57 ± 2.87

^a^SD: standard deviation

^b^Health-related quality of life

^c^Functional assessment of cancer therapy-breast version 4.0

^d^Connor–Davidson resilience scale

^e^Multidimensional scale of perceived social support

**Table 2:** Comparisons of resilience, social support, coping styles, and corresponding norms (*N* = 431)

	Score mean ± SD	Norm	MD^a^	95% CI^b^	*P*
*Resilience*		(*N* = 560)^e^			
CD-RISC^c^ total score	54.72 ± 14.71	65.4 ± 13.9	− 10.68	− 12.59, − 8.77	< 0.001
*Perceived social support*		(*N* = 1589)^f^			
MSPSS^d^ total score	65.45 ± 9.40	68.91 ± 11.99	− 3.46	− 4.68, − 2.24	< 0.001
*Coping style*		(*N* = 701)^g^			
Confrontation	19.87 ± 3.73	19.48 ± 3.81	0.39	− 0.10, 0.87	0.12
Avoidance	16.70 ± 1.90	14.44 ± 2.97	2.25	2.01, 2.50	< 0.001
Resignation	9.57 ± 2.87	8.81 ± 3.17	0.76	0.39, 1.13	< 0.001

^a^Mean difference

^b^Confidence interval

^c^Connor–Davidson resilience scale

^d^Multidimensional scale of perceived social support

^e^Adapted from: Davidson JRT. Connor–Davidson resilience scale (CD-RISC) Manual. Unpublished. 08–19-2018, accessible at www.cd-risc.com

^f^Adapted from: Jiang Q. Perceived social support scale. Chin J Behav Med Sci. 2001; 10(1): 41–43

^g^Adapted from: Shen X, Jiang Q. Report on application of Chinese version of MCMQ in 701 patients. Chin J Behav Med Sci. 2009; 9(1):18–20

After controlling for the socio-demographic and clinical characteristics, the three models showed different influences of resilience, PSS, and CS on HRQoL. Resilience (*B*s ranged from 0.64 to 0.75, *P* < 0.001), PSS (*B*s ranged from 0.22 to 0.29, *P* < 0.05), and CS (*B*s were 0.60 (confrontation) and − 1.68 (resignation), *P* < 0.05) significantly influenced HRQoL in model A and model C, with R^2^ of 0.64 and 0.66 (*P* < 0.001), respectively. In model B, only resilience [*B* = 0.81, 95%CI 0.66, 0.94, *P* < 0.001] and PSS [0.28 (0.05, 0.51), *P* = 0.016] significantly influenced HRQoL, with R^2^ of 0.64 (*P* < 0.001) (Table 3).

**Table 3:** Resilience, perceived social support, and coping style as independent predictors of health-related quality of life when controlling socio-demographic and clinical characteristics: multivariate linear regression analysis (*N* = 431)

Independent variables	*B* (95% CI)^d^	*P*
*Model A*^*a*^		
Resilience	0.75 (0.59, 0.92)	< 0.001
Perceived social support	0.29 (0.06, 0.51)	0.014
Coping style (confrontation)	0.60 (0.01, 1.19)	0.046
*Model B*^*b*^		
Resilience	0.81 (0.66, 0.97)	< 0.001
Perceived social support	0.28 (0.05, 0.51)	0.016
Coping Style (Avoidance)	0.82 (− 0.07, 1.71)	0.070
*Model C*^*c*^		
Resilience	0.64 (0.47, 0.81)	< 0.001
Perceived Social Support	0.22 (0.002, 0.45)	0.048
Coping Style (Resignation)	− 1.68 (− 2.48, − 0.89)	< 0.001

The independents were socio-demographics and clinical characteristics shown in Table 1: education level (ref. primary and below), marital status (ref. married), has children (ref. yes), employment status (ref. unemployed), average monthly income over the past 12 months (Chinese Yuan) (ref. < 3000), place of residence (ref. urban), chronic disease (ref. yes), illness stage (ref. 0–I), metastasis (ref. no metastasis), surgical style (ref. no surgery), adjuvant therapy (ref. conventional chemotherapy), and continuous characteristics (age)

^a^*R* = 0.80, *R*^2^ = 0.64, *F* = 14.33, *P* < 0.001

^b^*R* = 0.80, *R*^2^ = 0.64, *F* = 14.25, *P* < 0.001

^c^*R* = 0.81, *R*^2^ = 0.66, *F* = 15.76, *P* < 0.001

^d^confidence interval

**Fig. 2:**
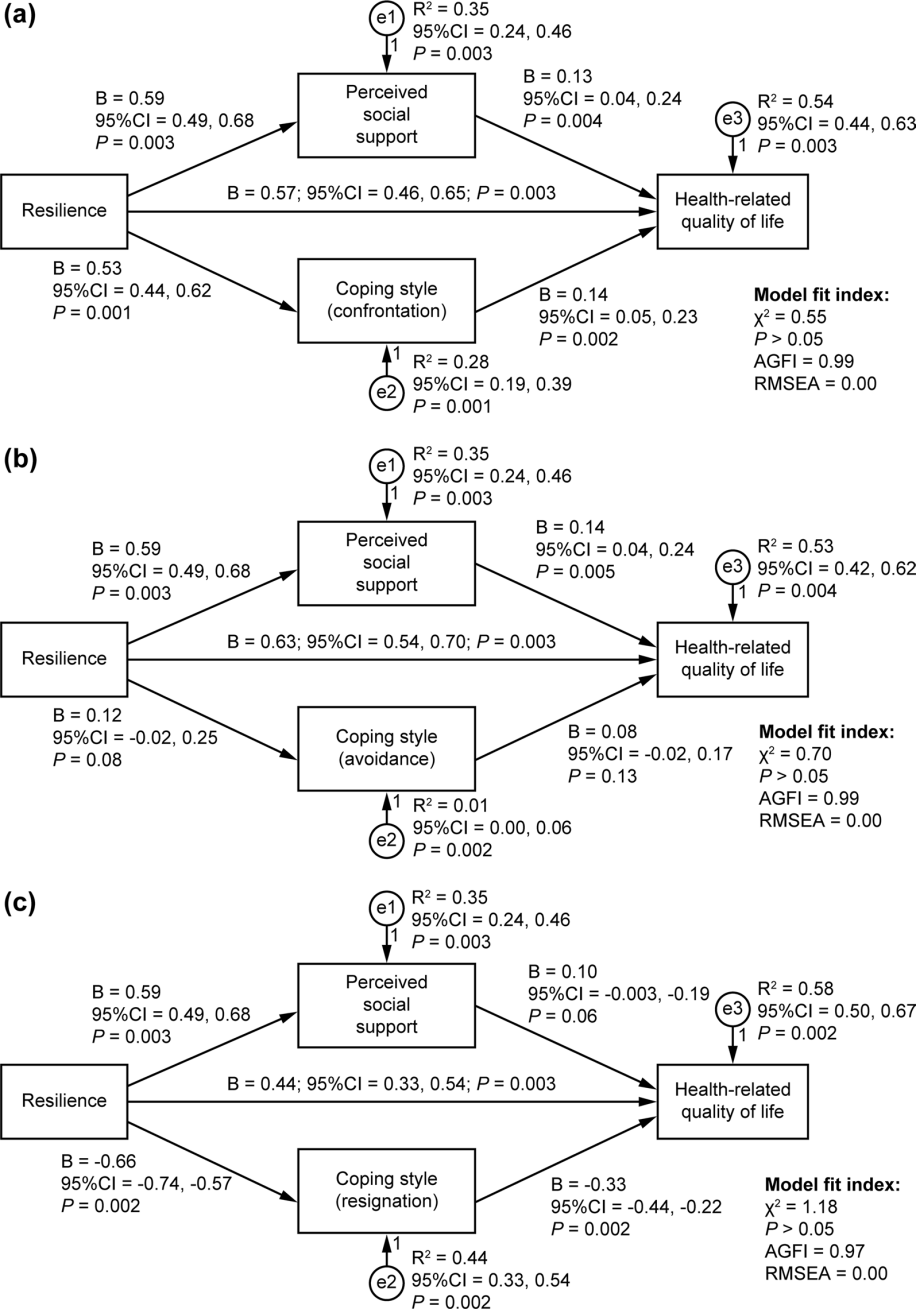
Mediator roles of perceived social support and coping style between resilience and health-related quality of life. Indirect effect: Model (**a**): *B* = 0.15, 95% CI [0.08, 0.23], *P* = 0.002; Model (**b**): *B* = 0.09, 95% CI [0.03, 0.15], *P* = 0.005; Model (**c**): *B* = 0.27, 95% CI [0.19, 0.36], *P* = 0.002. Total effect of each of the three models: *B* = 0.72, 95% CI [0.65, 0.78], *P* = 0.002. (AGFI: adjusted goodness-of-fit index; reference value: ≥ 0.90. RMSEA: root mean square error of approximation; reference value: < 0.08. χ^2^ value (desired significance *P* > 0.05). 95% CI 95% confidence interval. *B*: standardized regression weights.)

Figure 2 illustrates the SEM analysis that further showed resilience had significant direct effects on PSS (*B*s were 0.59, 95%CI 0.49, 0.68, *P* = 0.003), CS [*B*s were 0.53 (confrontation) and − 0.66 (resignation), *P* < 0.05], and HRQoL (*B*s ranged from 0.44 to 0.63, *P* < 0.05); resilience had significant indirect effects (*B*s ranged from 0.09 to 0.27, *P* < 0.05), and PSS (*B*s ranged from 0.13 to 0.14, *P* < 0.05) and CS [*B*s were 0.14 (confrontation) and − 0.33 (resignation), *P* < 0.05] had significant direct effects on HRQoL. The model fit indices were all satisfied (AGFI ≥ 0.90, RMSEA < 0.08, *P* > 0.05).

## Discussion

The findings show that women newly diagnosed with breast cancer had poor HRQoL, especially in the functional well-being. When comparing with the corresponding norm, the patients had lower level of resilience and PSS, and higher level of avoidance and resignation CSs. After controlling for socio-demographic and clinical characteristics, it demonstrates that HRQoL of the patients was significantly influenced by resilience, PSS, and confrontation and resignation CSs. Additionally, PSS and CS (confrontation and resignation) mediated the relationship between resilience and HRQoL.

The primary outcome HRQoL showed that the functional well-being had the lowest score among the five subscales, indicating that newly diagnosed women with breast cancer had the most prominent decrease in functional health. The emotional, social/family, and physical well-being subscales were also affected negatively during the disease course, demonstrating that the patients had difficulties with physical, psychological, and social functioning [5, 10]. The above poor health domains may be due to the adverse effects of the disease and treatment, especially the maladjustment during the rehabilitation process. Although the breast cancer-specific subscales for additional concerns had the highest scores among the five subscales, more attention should be devoted to the lower health domain scores in physical, social/family, emotional, and functional well-being that might be the result of concerns about potential future surgery and adjuvant therapy in clinical care for newly diagnosed patients [34, 35].

Of the secondary outcomes, the patients had lower level of resilience and PSS, and higher level of avoidance and resignation CSs than that of the corresponding norm. This is probably related to the condition that women newly diagnosed with breast cancer need more support to improve the ability of adaptation and positive coping with the disease. After closer inspection of the CD-RISC, MSPSS, and MCMQ subscales, we found that some patients were tenacious, perceived strong family support, and selected confrontation as their CS while dealing with the disease. It demonstrates that strong family support may be beneficial to improve the patient’s resilience, and the newly diagnosed patients may have great potential and power to improve their resilience, perceived social support, and positive coping style. Considering the significance of family networks in China, it is necessary to further focus on enhancing family resilience for the patients in breast cancer care [36, 37]. The psychosocial interventions should focus on improving poor resilience and enhancing positive CS of women newly diagnosed with breast cancer [38–40].

The findings that resilience, PSS, and confrontation and resignation CSs had significant influences on HRQoL partially support our first hypothesis that psychosocial factors are independent predictors of HRQoL [41–43], with higher resilience, perceived social support, and confrontation CS predicting better HRQoL, while resignation CS predicting poor HRQoL. This is probably due to the fact that a good psychological status such as strong resilience, PSS and positive CS is beneficial for a good health status [12–15]. The findings suggest that in HRQoL management programs, health care providers should pay more attention to the status of resilience, the level of perceived social support from family members and significant others, as well as which coping style do the newly diagnosed breast cancer patients used during the rehabilitation process. On this basis, a comprehensive and effective intervention could be developed for the HRQoL improvement. However, the avoidance CS did not significantly influence HRQoL. This may be because avoidance CS does not have a negative impact on HRQoL, since it is a preliminary protection for oneself to avoid psychological trauma temporarily until one is prepared in the face of a life event shock such as being newly diagnosed with breast cancer. Although it has been reported that negative CSs are negatively associated with HRQoL [44], the insignificant impacts of avoidance in women with newly diagnosed breast cancer in this study needs further examination.

The SEM analysis further identified the mediating roles of PSS and CS in the relationship between resilience and HRQoL. The findings support our second hypothesis that PSS and CS mediated the relationship between resilience and HRQoL, and indicate that higher PSS and the confrontation CS would strengthen the positive influence of resilience on HRQoL. Based on the Neuman Systems Model, we constructed the framework of the relationship among resilience (psychological defense), PSS and CS (personal response), and HRQoL (patient-reported outcomes). The findings further indicate that, except for the direct impact of resilience on HRQoL, a strong psychological defense would lead to positive personal response, and consequently achieve a better patient-reported outcome. Accordingly, interventions for improving PSS and enhancing the confrontation CS should be prioritized when developing HRQoL improvement programs. Given few studies reported the mediating roles of PSS and CS in the relationship between resilience and HRQoL in the similar samples of women with breast cancer [45–47], our findings provide new evidence for identifying the mediator roles of psychosocial factors in the relationship between resilience and HRQoL, and for developing multimodal intervention programs that consider PSS and CS to improve positive influences of resilience on HRQoL in women with newly diagnosed breast cancer.

This study has some limitations. First, the self-reported data of HRQoL, resilience, PSS, and CS lack objectivity. Information on related behaviors and physical health changes are recommended in future work. Second, the relationships among the above variables were possibly inflated due to response bias. Third, this cross-sectional study design could not support causal relationships among variables. Thus, their relationship trajectories during the long-term rehabilitation process should be further explored in longitudinal studies. Fourth, due to the non-simultaneity of the time of admission, the patients were recruited by convenience sampling, which might lead to potential bias and confounding. However, we tried our best to recruit every eligible breast cancer patient from the Tumor Hospital of Shaanxi Province and the First Affiliated Hospital of Xi’an Jiaotong University. Besides, we restricted the confounding of socio-demographic and clinical characteristics via multivariate linear regression analysis while analyzing the influences of resilience, PSS and CS on HRQoL. A multicenter cross-sectional survey with cluster sampling was recommended in future study design. Finally, the study was conducted in Xi’an, thus limiting the generalization of the findings to all women newly diagnosed with breast cancer. A multicenter design is recommended to enhance generalizability.

## Conclusions

The patients in our study had relatively poor HRQoL, especially in functional well-being with the lowest score of the FACT-Bv4.0 five subscales. In comparison with the corresponding norm, the patients were also found having lower resilience and PSS, and higher avoidance and resignation CSs. Resilience, PSS, and CS had significant direct effects on HRQoL, with PSS and CS mediating the influence of resilience on HRQoL. A multimodal intervention program focusing on PSS and CS needs to be developed to improve the positive influence of resilience on HRQoL in the breast cancer patient population.

## Data Availability

The datasets used and/or analysed during the current study are available from the corresponding author on reasonable request.

## Abbreviations

AGFI: Adjusted goodness-of-fit index
BCS: Breast cancer-specific
CD-RISC: Chinese Connor–Davidson resilience scale
CS: Coping style
EWB: Emotional well-being
FWB: Functional well-being
HRQoL: Health-related quality of life
MCMQ: Medical coping mode questionnaire
MSPSS: Multidimensional scale of perceived social support
NSM: Neuman systems model
PSS: Perceived social support
PWB: Physical well-being
RMSEA: Root mean square error of approximation
SEM: Structural equation modeling
SWB: Social/family well-being

## References

[1] Oh PJ, Cho JR (2020). Changes in fatigue, psychological distress, and quality of life after chemotherapy in women with breast cancer: a prospective study. Cancer Nurs.

[2] Shim E-J, Jeong D, Moon HG, Noh D-H, Jung S-Y, Lee E, Kim Z, Youn HJ, Cho J, Lee JE (2020). Profiles of depressive symptoms and the association with anxiety and quality of life in breast cancer survivors: a latent profile analysis. Qual Life Res.

[3] Montazeri A (2008). Health-related quality of life in breast cancer patients: a bibliographic review of the literature from 1974 to 2007. J Exp Clin Cancer Res.

[4] Mokhtari-Hessari P, Montazeri A (2020). Health-related quality of life in breast cancer patients: review of reviews from 2008 to 2018. Health Qual Life Outcomes.

[5] Hashemi S-M, Balouchi A, Al-Mawali A, Rafiemanesh H, Rezaie-Keikhaie K, Bouya S, Dehghan B, Farahani MA (2019). Health-related quality of life of breast cancer patients in the Eastern Mediterranean region: a systematic review and meta-analysis. Breast Cancer Res Treat.

[6] Siegel RL, Miller KD, Fuchs HE, Jemal A (2021). Cancer statistics, 2021. CA Cancer J Clin.

[7] Tu PC, Yeh DC, Hsieh HC (2020). Positive psychological changes after breast cancer diagnosis and treatment: the role of trait resilience and coping styles. J Psychosoc Oncol.

[8] Padilla-Ruiz M, Ruiz-Román C, Pérez-Ruiz E, Rueda A, Redondo M, Rivas-Ruiz F (2019). Clinical and sociodemographic factors that may influence the resilience of women surviving breast cancer: cross-sectional study. Support Care Cancer.

[9] Huang Y, Huang Y, Bao M, Zheng S, Du T, Wu K (2019). Psychological resilience of women after breast cancer surgery: a cross-sectional study of associated influencing factors. Psychol Health Med.

[10] Cho YU, Lee BG, Kim SH (2020). Coping style at diagnosis and its association with subsequent health-related quality of life in women with breast cancer. Eur J Oncol Nurs.

[11] Mokhtari L, Markani AK, Khalkhali HR, Feizi A (2022). The perceived social support by Iranian women with breast cancer: a qualitative study. Support Care Cancer.

[12] Edward KL, Chipman M, Giandinoto JA, Robinson K (2019). Quality of life and personal resilience in the first two years after breast cancer diagnosis: systematic integrative review. Br J Nurs.

[13] Oh GH, Yeom C-W, Shim E-J, Jung D, Lee K-M, Son K-L, Kim W-H, Moon JY, Jung S, Kim T-Y, Im S-A, Lee K-H, Hahm B-J (2020). The effect of perceived social support on chemotherapy-related symptoms in patients with breast cancer: a prospective observational study. J Psychosom Res.

[14] Mishra VS, Saranath D (2019). Association between demographic features and perceived social support in the mental adjustment to breast cancer. Psychooncology.

[15] Gall TL, Bilodeau C (2020). The role of positive and negative religious/spiritual coping in women’s adjustment to breast cancer: a longitudinal study. J Psychosoc Oncol.

[16] Popa-Velea O, Diaconescu L, Jidveian Popescu M, Trutescu C (2017). Resilience and active coping style: effects on the self-reported quality of life in cancer patients. Int J Psychiatry Med.

[17] Ruiz-Rodriguez I, Hombrados-Mendieta I, Melguizo-Garin A, Martos-Mendez MJ (2022). The importance of social support, optimism and resilience on the quality of life of cancer patients. Front Psychol.

[18] Zhang H, Zhao Q, Cao P, Ren G (2017). Resilience and Quality of Life: exploring the mediator role of social support in patients with breast cancer. Med Sci Monit.

[19] Neuman B (1996). The Neuman systems model in research and practice. Nurs Sci Q.

[20] Yan H (2015). Medical statistics.

[21] Wan C, Zhang D, Yang Z, Tu X, Tang W, Feng C, Wang H, Tang X (2007). Validation of the simplified Chinese version of the FACT-B for measuring quality of life for patients with breast cancer. Breast Cancer Res Treat.

[22] Brady MJ, Cella DF, Mo F, Bonomi AE, Tulsky DS, Lloyd SR, Deasy S, Cobleigh M, Shiomoto G (1997). Reliability and validity of the functional assessment of cancer therapy-breast quality-of-life instrument. J Clin Oncol.

[23] Yu XN, Lau JT, Mak WW, Zhang J, Lui WW, Zhang J (2011). Factor structure and psychometric properties of the Connor-Davidson Resilience Scale among Chinese adolescents. Compr Psychiatry.

[24] Connor KM, Davidson JR (2003). Development of a new resilience scale: the connor-davidson resilience scale (CD-RISC). Depress Anxiety.

[25] Chen Y, Ma H, Chen Z, Jia Y, Wang X, Chen J (2018). Reliability and validity of Chinese version of multidimensional scale of perceived social support in elderly people with chronic disease. J Nurs.

[26] Zimet GD, Powell SS, Farley GK, Werkman S, Berkoff KA (1990). Psychometric characteristics of the multidimensional scale of perceived social support. J Per Assess.

[27] Guan NC, Seng LH, Hway AY, Hui KO (2015). Factorial validity and reliability of the Malaysian simplified Chinese version of Multidimensional Scale of Perceived Social Support (MSPSS-SCV) among a group of university students. Asia Pac J Public Health.

[28] Zhou K, Li H, Wei X, Yin J, Liang P, Zhang H, Kou L, Hao M, You L, Li X, Zhuang G (2015). Reliability and validity of the multidimensional scale of perceived social support in Chinese mainland patients with methadone maintenance treatment. Compr Psychiatry.

[29] Shen X, Jiang Q (2000). Report on application of Chinese version of MCMQ in 701 patients. Chin J Behav Med Sci.

[30] Feifel H, Strack S, Nagy VT (1987). Degree of life-threat and differential use of coping style. J Psychosom Res.

[31] Zhang P (2011). Statistical analysis based on bootstrap method. J Yibin Univ.

[32] Baron RM, Kenny DA (1986). The moderator-mediator variable distinction in social psychological research: conceptual, strategic, and statistical consideration. J Pers Soc Psychol.

[33] Wu ML (2010). Structural equation modeling: AMOS operation and application.

[34] Zhou K, Wang W, An J, Li M, Li J, Li X (2019). Effects of progressive upper limb exercises and muscle relaxation training on upper limb function and health-related quality of life following surgery in women with breast cancer: a clinical randomized controlled trial. Ann Surg Oncol.

[35] Zhou K, Wang W, Zhao W, Li L, Zhang M, Guo P, Zhou C, Li M, An J, Li J, Li X (2020). Benefits of a WeChat-based multimodal nursing program on early rehabilitation in postoperative women with breast cancer: a clinical randomized controlled trial. Int J Nurs Studs.

[36] Li Y, Qiao Y, Luan X, Li S, Wang K (2019). Family resilience and psychological well-being among Chinese breast cancer survivors and their caregivers. Eur J Cancer Care.

[37] Li Y, Wang K, Yin Y, Li Y, Li S (2018). Relationships between family resilience, breast cancer survivors’ individual resilience, and caregiver burden: a cross-sectional study. Int J Nurs Stud.

[38] Wu Z, Liu Y, Li X, Li X (2016). Resilience and associated factors among Mainland Chinese women newly diagnosed with breast cancer. PLoS ONE.

[39] Lam WW, Bonanno GA, Mancini AD, Ho S, Chan M, Hung WK, Or A, Fielding R (2010). Trajectories of psychological distress among Chinese women diagnosed with breast cancer. Psychooncology.

[40] Li L, Li S, Wang Y, Yi J, Yang Y, He J, Zhu X (2017). Coping profiles differentiate psychological adjustment in Chinese women newly diagnosed with breast cancer. Integr Cancer Ther.

[41] Wang Y, Zhao Y, Xie S, Wang X, Chen Q, Xia X (2019). Resilience mediates the relationship between social support and quality of life in patients with primary glaucoma. Front Psychiatry.

[42] Wu M, Yang Y, Zhang D, Zhao X, Sun Y, Xie H, Jia J, Su Y, Li Y (2018). Association between social support and health-related quality of life among Chinese rural elders in nursing homes: the mediating role of resilience. Qual Life Res.

[43] Pan CJ, Liu HC, Liang SY, Liu CY, Wu WW, Cheng SF (2019). Resilience and coping strategies influencing the quality of life in patients with brain tumor. Clin Nurs Res.

[44] Toscano A, Blanchin M, Bourdon M, Bonnaud Antignac A, Sebille V (2020). Longitudinal associations between coping strategies, locus of control and health-related quality of life in patients with breast cancer or melanoma. Qual Life Res.

[45] Wang Z, Xu J (2017). Association between resilience and quality of life in Wenchuan earthquake Shidu parents: the mediating role of social support. Commun Ment Health J.

[46] Lee JH, Kim HY (2018). Symptom distress and coping in young Korean breast cancer survivors: the mediating effects of social support and resilience. J Korean Acad Nurs.

[47] Dooley LN, Slavich GM, Moreno PI, Bower JE (2017). Strength through adversity: moderate lifetime stress exposure is associated with psychological resilience in breast cancer survivors. Stress Health.

